# Alteration of Zeta potential and membrane permeability in bacteria: a study with cationic agents

**DOI:** 10.1186/s40064-015-1476-7

**Published:** 2015-11-04

**Authors:** Suman Halder, Kirendra Kumar Yadav, Ratul Sarkar, Sudipta Mukherjee, Pritam Saha, Saubhik Haldar, Sanmoy Karmakar, Tuhinadri Sen

**Affiliations:** Division of Pharmacology, Department of Pharmaceutical Technology, Jadavpur University, Kolkata, 700032 West Bengal India; Department of Chemistry, Jadavpur University, Kolkata, 700032 India

**Keywords:** Cell viability, Membrane permeability, Polydispersity index (PDI), Zeta potential

## Abstract

In the present study, we have tried to establish the correlation between changes in Zeta potential with that of cell surface permeability using bacteria (*Escherichia coli* and *Staphylococcus aureus*). An effort has been made to establish Zeta potential as a possible marker for the assessment of membrane damage, with a scope for predicting alteration of cell viability. Cationic agents like, cetyl trimethyl ammonium bromide and polymyxin B were used for inducing alteration of Zeta potential, and the changes occurring in the membrane permeability were studied. In addition, assessment of poly-dispersity index (PDI), cell viability along with confocal microscopic analysis were performed. Based on our results, it can be suggested that alteration of Zeta potential may be correlated to the enhancement of membrane permeability and PDI, and it was observed that beyond a critical point, it leads to cell death (both Gram-positive and Gram-negative bacteria). The present findings can not only be used for studying membrane active molecules but also for understanding the surface potential versus permeability relationship.

## Background

In drug discovery and development, targeting the bacterial cell surface is now an emerging area, considering the escalating number of evidences of multiple drug resistant pathogens and a gradually decreasing availability of antibiotics (Hurdle et al. 2011). Despite persistence of some unfavorable situations (cross targeting of the mammalian cell membrane by bacterial membrane acting agents) (Payne et al. 2007), targeting the bacterial surface seems to be of paramount interest because the surface acting agents have been found to display remarkable bactericidal effect and simultaneously demonstrate lesser tendency to trigger resistance development (Hurdle et al. 2011; Bambeke et al. 2008). According to reports, the envelope of a bacterial cell behaves as a chemical compartment (Hurdle et al. 2011; Torcato et al. 2012), helping to maintain cellular homeostasis (Sondi and Salopek-Sondi 2005), thereby playing a significant role in maintaining diverse physiological functions (Herben et al. 1990; Urrutia-Mera et al. 1992; Clark et al. 1999; Wilson et al. 2001; Eboigbodin et al. 2006) and also acting as a barrier, providing selective permeability. Irrespective of cell wall behavior (Gram-positive or Gram-negative), bacterial surfaces possess acidic and basic functional groups that are known to be associated with lipopolysaccharides (LPS), phospholipids (in case of Gram-negative bacteria) or peptidoglycan, teichoic acid (in case of Gram-positive bacteria) (Yongsuk and Brown 2006). The presence of such functional groups influence the electrostatic behavior (Yongsuk and Brown 2008) of the cells, thus regulating the bacterial adhesion (Takashima and Morisaki 1997; Jucker et al. 1997; Sharma and Rao 2003; Loosdrecht et al. 1987; Yoshinari et al. 2000; Walker et al. 2005; Chen and Walker 2007) and also contribute towards interaction with various agents (Scholl and Harvey 1992; Yee et al. 2000; Mills et al. 1994; Borrok et al. 2004).

Considering the emerging importance of membranes (dissipation of membrane potential) as targets for antimicrobial therapy, and based on the available information from different membrane targeting drugs (nisin, alamethicin, valinomycin, etc.), it may be mentioned that these drugs either target the Min proteins (associated with cell division) or may affect the cell morphology, thereby causing alteration of membrane potential with subsequent changes in the morphogenic proteins (Strahl and Hamoen 2010).

Bacterial surface charge has often been described by the Zeta potential, an electrochemical property of the cell surface, which represents the potential at the shear plane of the electrical double layer encompassing a cell in solution (Tokumasu et al. 2012; Soon et al. 2011). In general, for most bacteria, the net surface charge is negative and is balanced by oppositely charged counter ions present in the surrounding media (Cieśla et al. 2011). Zeta potential (the electrical potential difference at the hydrodynamic slipping surface that may be described as the interface between the aqueous liquid and the stationary layer of fluid adhering to the bacterial cell surface), is known to play a significant role towards maintenance of the cellular function and also provides useful information about cell surface characteristics (Tokumasu et al. 2012; Saito et al. 2001). At times, the interaction between the bacterial surface and various agents may be governed by electrostatic interactions, which in turn may affect Zeta potential, which may subsequently alter cell surface permeability leading to cell death. It has also been observed that alteration of erythrocyte Zeta potential (achieved through increased dielectric constant or by changing the composition of the medium) may often lead to agglutination (Fernandes et al. 2011). Thus further research on membrane acting molecules (particularly with antimicrobial properties) would be of enormous importance considering the gradual shrinkage of the antibiotic pipeline.

The work was carried out to establish a relationship between altered surface potential of the bacteria (represented as Zeta potential) with that of cell surface permeability and poly dispersity index, following exposure to surface acting agents. Hence, in the present study, the cells (Gram-positive as well as Gram-negative bacteria) were treated with surface active agents [cetyl trimethyl ammonium bromide (CTAB) and polymyxin B] and also with ampicillin, an antiobiotic, known to work through inhibition of cell wall biosynthesis. Thus an attempt was made to understand the role of these agents on cell membrane architecture, using Zeta potentials a tool for studying the alteration in bacterial cell surface permeability and subsequent bacterial viability.

## Results and discussion

In the present study, *E. coli* and *S. aureus* were exposed to different concentration of CTAB (0.3–170 µg/ml), and polymyxin B (0.3–170 µg/ml) and the treated cells were examined for any possible alteration of cell surface permeability along with corresponding alteration in Zeta potential. The alteration of cell surface permeability was confirmed by NPN and crystal violet assay, performed under identical experimental conditions. In the present research work we have selected a range of concentration, along with different time intervals 30, 60, 90 and 120 min for studying the effect of both concentration as well as time of exposure, for a better understanding of the drug action.

According to Balhara et al. (2013), the interaction of membrane acting agents with a cell surface may involve variety of mechanisms, namely interaction of different functional groups with bacterial surface or aggregation within the membrane, resulting in the perturbation of membrane integrity. As evident from studies with antimicrobial peptides, mechanism of membrane active agents involve the formation of—barrel-stave, carpet, toroidal-pore, or aggregate channel models (Giuliani et al. 2008; Nguyen et al. 2011; Sato and Feix 2006; Alves et al. 2010; Li et al. 2012), which leads to increase in cell permeability, which may ultimately result in cell death (Powers and Hancock 2003).

### Zeta potential

**Fig. 1:**
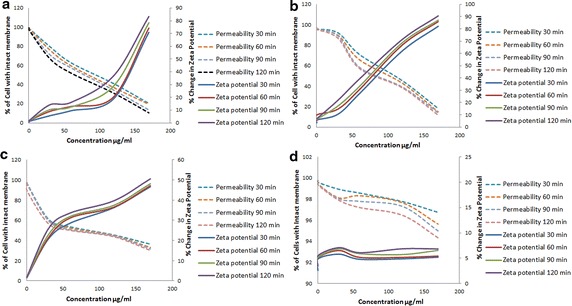
Change in bacterial (**a**, **c** *E. coli*: **b**, **d** *S. aureus*) Zeta potential and membrane permeability (assayed by NPN uptake) in presence of different concentrations of CTAB (**a**, **b**) and polymyxin B (**c**, **d**). Percentage change in Zeta potential (*solid lines*) and percentage change in permeability (*dashed lines*) was plotted against the concentration (µg/ml) of the treatment

**Fig. 2:**
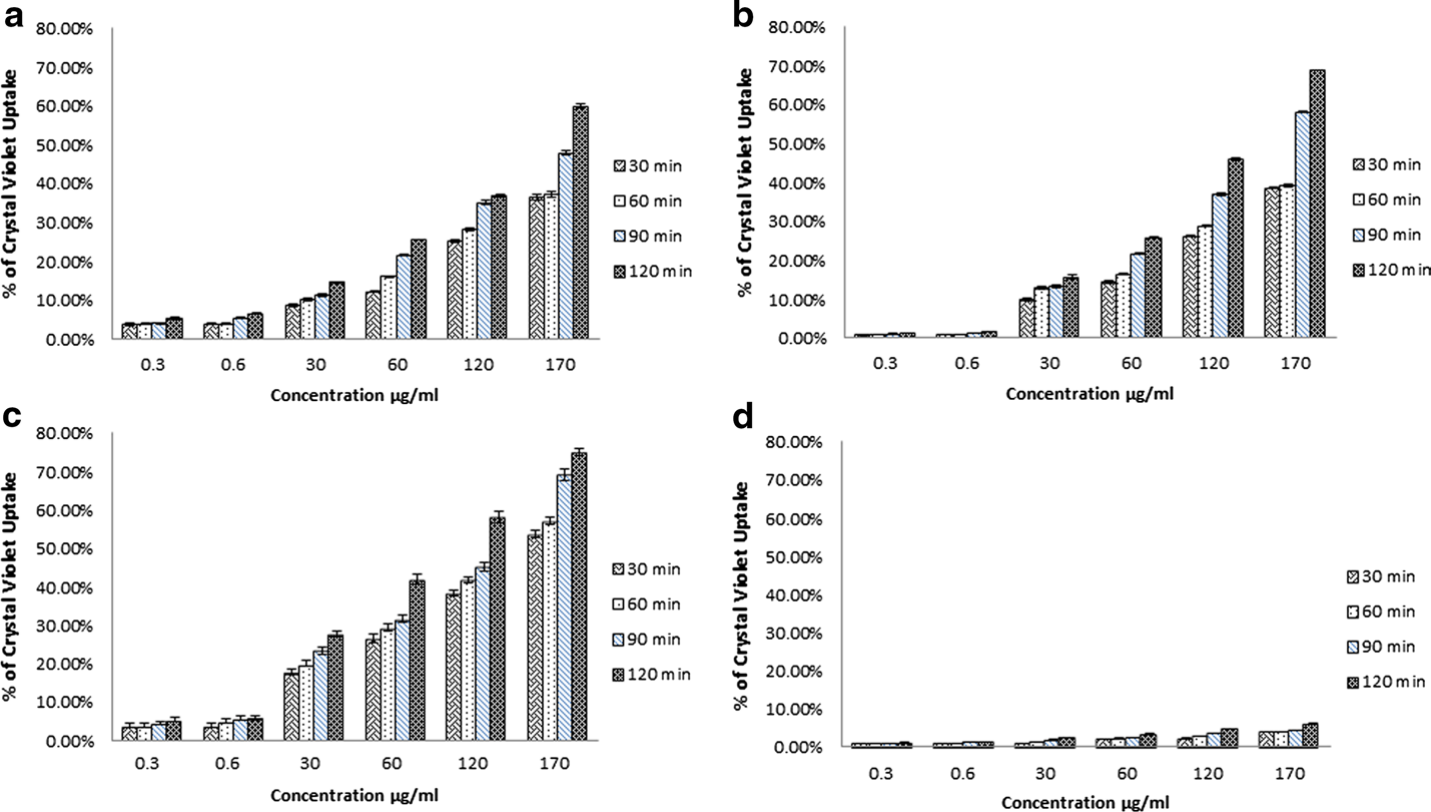
Change in bacterial (**a**, **b** *E. coli*; **c**, **d** *S. aureus*) membrane permeability (assayed by crystal violet uptake) in presence of different concentrations of CTAB (**a**, **c**) and polymyxin B (**b**, **d**). Percentage of crystal violet uptake was plotted against the concentration (µg/ml) of the treatment

Surface neutralisations of the membrane are important for the antimicrobial activity of the certain substances, which acts on bacterial surface (Torcato et al. 2013). In our study, the average Zeta potential of the untreated *E. coli* and *S. aureus* were found to be −44.2 and −35.6 mV, respectively. It may be mentioned that the presence of additional layer of negatively charged LPS in Gram-negative bacteria as compared to Gram-positive bacteria, has been attributed to the higher negative potential of *E. coli* than that of *S. aureus*, and our observation were found to be similar to the previous findings (Arakha et al. 2015; Alves et al. 2010; Domingues et al. 2014). As evident from our results, CTAB (0.3, 0.6 µg/ml) did not produce any alteration of the Zeta potential (up to 2 h) in either *E. coli* or *S. aureus* (Fig. 1a, b). However, a alteration of Zeta potential could be observed in both *E. coli* and *S. aureus*, when the cells were exposed to CTAB, at concentrations of 30 µg/ml and above (Fig. 1a, b). Moreover, it was also noticed that in case of CTAB, the magnitude of decrease in the negativity of the Zeta potential was found to be greater at higher concentrations and such alteration of Zeta potential was found to be time dependent (studied till 2 h) as well. Similar findings were also recorded when bacterial cell suspension was treated with ZnO nanoparticle, where positive surface potential of ZnO nanoparticles were found to interact with the negative surface potential of bacterial membrane and the potential shifted towards neutrality and this behavior was found to be dependent on the concentration of ZnO nanoparticles and resulted in the destabilization of the membrane (Arakha et al. 2015). Interestingly, such change in potential (with CTAB) can be correlated with the increased membrane permeability as was evident from NPN and crystal violet assay (Figs. 1, 2). The present observations are in conformity with earlier reports, where it has been stated that surface charge neutralization leads to altered membrane permeability (Alves et al. 2010). Another important finding of this present investigation is related to the decrease in the negativity of the Zeta potential that was found to be more in *S. aureus* than in *E. coli*, when studied at identical concentration (Fig. 1). Therefore, such alteration of Zeta potential in Gram-negative bacteria may be caused due to the presence of higher density of anionic groups and O-antigen in LPS membrane, apart from the thin peptidoglycan layer, which also acts as an additional barrier for the solutes, thus helping in the maintenance of the surface potential and in turn membrane integrity (Domingues et al. 2014).

According to reports, the cyclic moiety of Polymyxin B may be responsible for LPS mediated membrane association, with subsequent penetration into the inner bilayer core, thereby possibly producing alteration in membrane permeability (Katz et al. 2003) through displacement of Ca^2+^ and Mg^2+^ ions and this results in the rupture of the cell membrane (Mendes and Burdmann 2010). In another study performed with ovotransferrin, an antimicrobial peptide (OTAP-92), the compound was found to interact with lipid bilayers and subsequently alter the membrane potential. Addition of OTAP-92 (bactericidal concentration) to hyperpolarized liposomes also caused leakage of the dye (positively charged) due to depolarization of the vesicles (Ibrahim et al. 2000).

According to our observations, Polymyxin B produced alteration of the magnitude of Zeta potential in Gram-negative cells (*E. coli*) but failed to produce any alteration in *S. aureus* (Fig. 1c, d). A similar finding was also observed with Gram-negative *Acinetobacter baumannii* and also with artificial bio-membrane model, following exposure to polymyxin B (Soon et al. 2011; Domingues et al. 2012). It may be mentioned that the LPS barrier in Gram-negative bacteria was found to be destabilized at physiological pH, by electrostatic attraction of the cationic polymyxin B, leading to permeabilsation of the membrane structure (Kennedy et al. 2011; Soon et al. 2011; Domingues et al. 2012).

According to Ahn et al. (2001), use of cationic substances like aluminium affects the surface negativity (Zeta potential) of membranes (root cells), leading to alteration of lipid mediated signaling and membrane destabilization. It may be pertinent to mention that these cations interfere with the functioning of membrane bound ATPase (responsible for maintaining membrane potential through regulation of H^+^ movement across the membrane). In situations of cation-induced toxicity, Zeta potential could be considered as an important parameter for governing membrane damage, particularly associated with decreased membrane potential (resulting in decreased ATPase activity).

### Polydispersity index and viability of the bacterial cells

In Table 1, we have depicted the relationship between cell viability (measured by counting colony-forming units) and polydispersity index (PDI) of the bacterial suspension, following exposure to different concentration of CTAB. Exposure of the bacterial cells to the disrupting agents causes rupture in the cell surface which may lead to cell death, as evident from significant reduction of cell viability (decreased CFU). The rupturing of the bacterial cell surface significantly increased the total particulate content of the cell suspension (Table 1; treatment with CTAB—30 µg/ml), as was observed in our study. However, lower concentration of CTAB (0.3 µg/ml) neither decreased the CFU nor the PDI (measured at 2 h) in either *E. coli* or *S. aureus*.

**Table 1:** The polydispersity index (measured by DLS) and viability of cells (expressed as CFU) after treatment with and without CTAB

Treatment	log(CFU)		Polydispersity index	
	*E. coli*	*S. aureus*	*E. coli*	*S. aureus*
Normal bacterial cell	9.14 ± 0.10	8.41 ± 0.24	0.31 ± 0.019	0.28 ± 0.023
Cell treated with 0.3 µg/ml CTAB	9.04 ± 0.52	8.05 ± 0.35	0.32 ± 0.032	0.29 ± 0.016
Cell treated with 30 µg/ml CTAB	5.06 ± 0.52**	4.02 ± 0.44**	0.75 ± 0.023**	0.79 ± 0.014**

Values are expressed as mean ± SEM; (n = 6)

** p < 0.01 (vs. control)

### NPN assay

The outer membrane (OM) of Gram-negative bacteria, in particular, is known to be impervious to different hydrophobic chemical agents, including NPN, owing to the presence of the LPS, present on the outer side (Nikaido 1996). However, permeabilising agents (which destabilizes the OM and liberate the OM components or intercalate with the OM), have been found to alter the uptake of substances like NPN, thus making it an effective marker for studying cell permeability. Permeability studies, particularly when performed on Gram-negative microbes, could be of importance particularly in relation to antimicrobial drug development. The ability of any agent, causing damage to the outer cell surface has been extensively quantified by NPN assay. As NPN is hydrophobic in nature, hence it cannot penetrate through intact membranes and therefore exhibits weak fluorescence emission in a buffer medium, whereas an enhanced uptake of NPN (increased fluorescence) may occur in case of damaged (functionally invalid) outer membranes (Loh et al. 1984; Torcato et al. 2013).

In the present context, the NPN uptake was found to be negligible (till 2 h) for the untreated *E. coli* or *S. aureus*, thereby indicating the existence of intact cell surface (data not shown). Addition of CTAB (30–170 µg/ml) to either *E. coli* or *S. aureus* (in the presence of NPN), produced a concentration dependent as well as time dependent enhancement in fluorescence emission (indication of NPN uptake). The positive charge of CTAB binds electrostatically bond with negatively charged LPS of Gram-negative cell surface, and also with the negatively charged teichoic acid, present in Gram-positive cells, and such interaction may create stress, leading to enhancement of cell permeability (Simões et al. 2005; Domingues et al. 2014; Berry et al. 2005; Maillard 2002). Moreover, the magnitude of alteration of fluorescence emission was higher in case of *S. aureus* than that of *E. coli*. Interestingly, higher change in NPN uptake was observed with CTAB pretreated Gram-positive *S. aureus*, where the change in the Zeta potential was also found to be more (Fig. 1a, b) as compared to CTAB treated *E. coli*, thereby indicating higher effectiveness of CTAB against Gram-positive cells as compared to the Gram-negative ones and this may be due to the distinct differences of their cell surface architecture.

Polymyxin B produced a concentration dependent increase in fluorescent emission in case of *E. coli*, which is in sharp contrast to that of *S. aureus*, (Fig. 1c, d). Therefore, polymyxin B was found to alter both Zeta potential and cell permeability in Gram-negative bacteria (Fig. 1c), but was found to be ineffective in Gram-positive cells (Fig. 1d) and our observation are in conformity with the findings of Wiese et al. (1998), where a detergent like mechanism of Polymyxin B has been attributed to its membrane interaction (lesion formation) properties. Substances like polymyxin B is known to display electrostatic interaction with anionic LPS, leading to alteration of cell membrane architecture, thus enhancing permeability, leakage of cell components and subsequent cell death (Falagas and Kasiakou 2005).

### Measurement of permeability with crystal violet

Hydrophobic crystal violet has been known to display weak penetration of the outer membrane but on the contrary, it has been found to penetrate cells with impaired cell membranes thus, crystal violet assay may be employed for the detection of membrane damage and such study with this dye may provide useful information regarding altered membrane permeability (Devi et al. 2010; Tsuchido et al. 1985). In this study, CTAB was found to exhibit a concentration dependent as well as time dependent augmentation in the uptake of crystal violet (Fig. 2a, c), moreover, the extent of crystal violet uptake was always found to be higher in *S. aureus* than that of *E. coli* (Fig. 2a, c).

Polymyxin B produced significant uptake of crystal violet in Gram-negative cells and this enhancing effect was also found to be both concentration as well as time dependent (Fig. 2b). However, polymyxin B didnot exhibit any remarkable variation of crystal violet uptake in *S. aureus*, (Fig. 2d).

In order to ascertain the relationship between the altered membrane permeability with that of Zeta potential, two additional approaches were undertaken, where both *E. coli* and *S. aureus* were exposed to (1) different concentration of Ampicillin and (2) exposed to heat treatment (100 °C for 10 min) and the cells were examined for NPN uptake and the Zeta potential was also analysed.

**Fig. 3:**
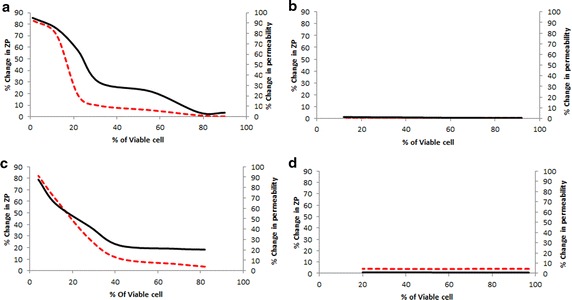
*E. coli* (**a**, **b**) and *S. aureus* (**c**, **d**) cells were treated with different concentrations of CTAB (**a**, **c**) and ampicillin (**b**, **d**). Viability of the cells is the common parameter which has changed in a dose dependent manner for both of the agents, thus percentage change in Zeta potential (ZP; *dashed red line*) and percentage change in permeability (*solid black line*) was plotted against percentage of viable cells

Ampicillin (a penicillin derivative) acts as an irreversible inhibitor of trans-peptidase, an enzyme responsible for the formation of the bacterial cell wall (Noller et al. 2000). From Fig. 3, it was evident that the drug (concentration) neither affected the zeta potential nor the membrane permeability (NPN assay), however it reduced the cell viability. As discussed earlier, treatment of both *E. coli* and *S. aureus* with CTAB produced alteration of Zeta potential and it could be correlated to membrane disruption associated reduction in cell viability (Fig. 3).

Heat treatment (10 min) was found to alter both Zeta potential as well as membrane permeability (NPN uptake) in *E. coli* but no such change could be observed in the Gram-positive cells. However, on increasing the heat exposure to 30 min, the Zeta potential of both *E. coli* and *S. aureus* were found to decrease and there was a significant enhancement of NPN uptake in both the cell types, when compared to 10 min exposure (Table 2). In earlier studies, it has been observed that increasing the temperature (around 80 °C) increases membrane depolarization coupled with enhanced permeability (Kennedy et al. 2011). However, such resistance to change (10 min exposure), as observed with *S. aureus* (Table 2) could be attributed to the presence of the thick peptidoglycan layer in Gram-positive bacteria.

**Table 2:** Zeta potential (mV) and mean fluorescence intensity of normal, heat treated and autoclaved *E. coli* and *S. aureus*

Treatment	Zeta potential (mV)		Fluorescence intensity (NPN uptake)	
	*E. coli*	*S. aureus*	*E. coli*	*S. aureus*
Normal bacterial cell	−44.2 ± 0.50	−35.6 ± 0.54	975 ± 55.49	855 ± 60.83
Heat treatment for 10 min at 100 °C	−33.9 ± 0.81**	−35.13 ± 0.60	1248 ± 138.82**	872 ± 62.96
Heat treatment for 30 min at 100 °C	−21.1 ± 0.62**	−27.6 ± 0.54**	1543 ± 76.01**	1209 ± 70.34**

Values are expressed as mean ± SEM; (n = 6)

The respective values of Zeta potential and NPN assay of normal, heat treated and autoclaved *E. coli* and *S. aureus*

The data are the means ± the standard deviations (n = 6)

** p < 0.01. (vs. control)

**Fig. 4:**
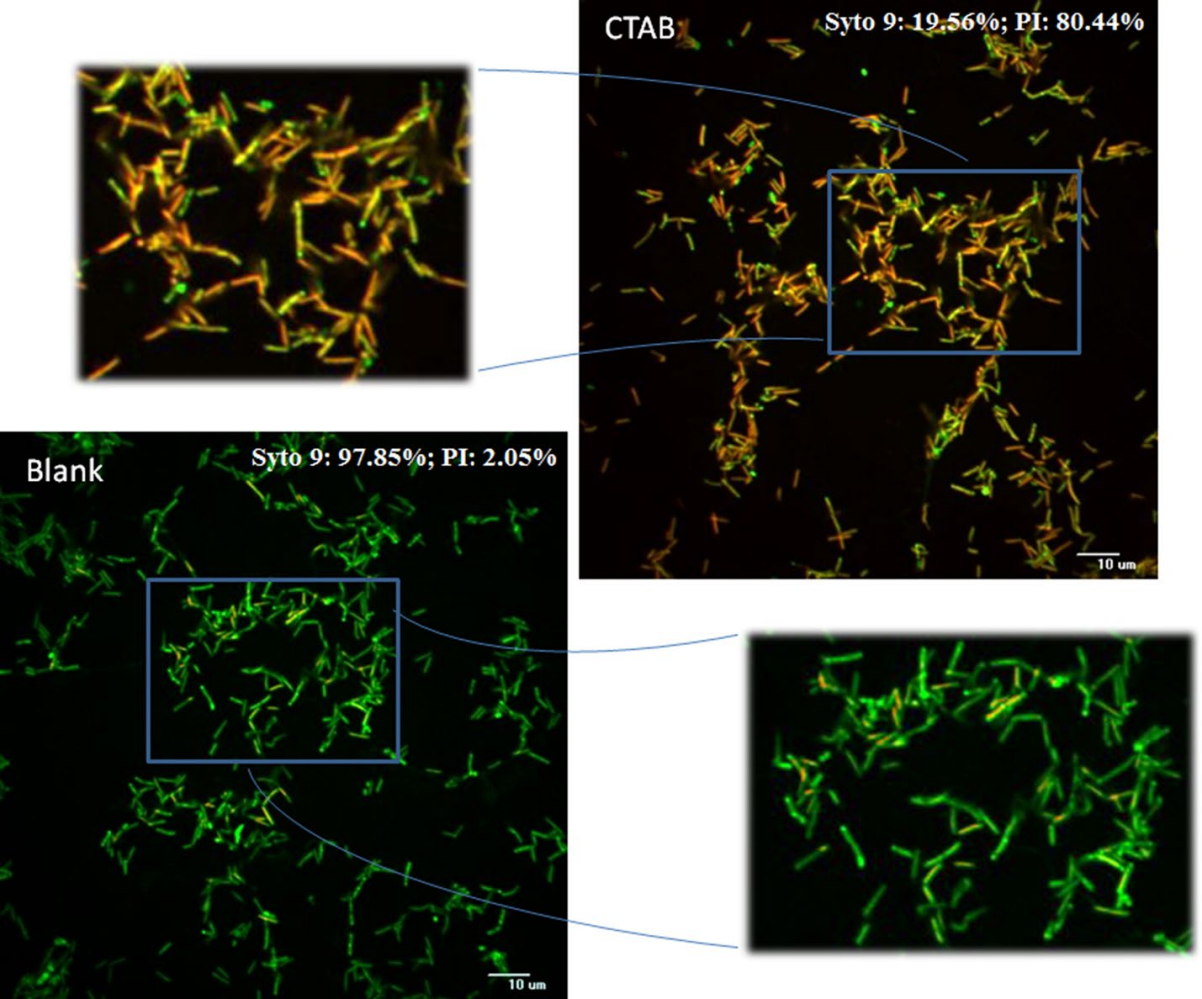
Bacterial cell suspension (CTAB 30 µg/ml and untreated Blank) stained with Syto-9 and PI analysed by CLSM. Cells with intact cell surface (membrane undisturbed) stained fluorescent *green*, whereas cells with ruptured surface (altered membrane integrity) stained fluorescent *red*. The overlap of the *red* and *greens* appear as *orange*

The alteration of cell viability following CTAB pre-treatment was further confirmed by CLSM, and from Fig. 4 it was evident that CTAB pre-treatment increased the density of PI stained (red) cells as compared to the blank, confirming the reduction of cell viability, a consequence of enhanced cell membrane permeability (evident from crystal violet and NPN assay), possibly caused due to alteration of Zeta potential. From our study it was further observed that 80.44 % of the cells could be stained by PI following treatment with CTAB, whereas only 2.05 % cells could be stained with PI in the blank.

## Conclusion

For a cell to survive, it needs to maintain the membrane architecture for the purpose of regulating the trans-membrane potential, as these are essential prerequisites for growth as well as metabolic activity. Based on these preliminary studies, we observed that the surface acting agents produced an alteration of Zeta potential (both types of the bacterial strains) along with increased surface permeability (used as a marker for membrane permeability), thereby revealing a possible correlation between these two, which can eventually be linked to decreased cell viability. Considering the importance of membrane targeting in contemporary antibacterial drug discovery, the present study helps us to establish a correlation between altered Zeta potential and decreased cell viability (caused due to the membrane disruption). Moreover, it may also be pertinent to state that altered membrane depolarization may not always lead to death, rather depending on the degree of alteration; the functioning of the cell may be affected. Studies related to membrane depolarization (based on the Zeta potential values), could be of importance as it would help to indicate the sensitizing (permeabilizing property) effect of the compound, which have been observed to be valid even at non toxic concentrations. Therefore, permeabilizing agents may be useful for combining with other agents to improve the uptake of antimicrobial molecules. Nevertheless, establishing a critical point (Zeta potential), could be also useful for prediction of cell death. Therefore, based on the present findings, future modification of the laser technology (DLS) could make Zeta potential as a useful probe for studying drug-membrane interactions. Furthermore, being rapid as well as straightforward and inexpensive, the technique can be adopted for screening of molecules with membrane perturbing properties.

## Methods

### Bacterial strains

The test microorganisms utilized in this study included both Gram-positive (*Staphylococcus aureus* MTCC 96) and Gram-negative bacteria (*Escherichia coli* MTCC *2939*). The strains were maintained on nutrient agar (NA) plate and were stored at 4 °C. A single isolated colony was picked from this plate and transferred to Mueller–Hinton Broth (MHB) and was incubated at 37 °C. Density of the broth was adjusted to 0.5 McFarland standard with MHB (Andrews 2001).

### Chemicals

Chemicals utilized in the present investigation were CTAB (Merck), polymyxin B (Hi-media), crystal violet, 1-*N*-phenylnaphthylamine (NPN) (Hi-media), gentamicin sulphate (Hi-media), ampicillin sodium salt (Hi-media), potassium phosphate dibasic anhydrous (K_2_HPO_4_) (SRL) and potassium di-hydrogen phosphate (KH_2_PO_4_) (Merck). All other chemicals utilized in this study were of analytical grade, purchased from Sigma Aldrich.

### Preparation of stock solution

Stock solution of 10 mg/ml of CTAB, polymyxin B and ampicillin were prepared individually in 0.5 mM potassium phosphate buffer solution (pH7.4). Different concentration of CTAB (0.3, 0.6, 30, 60, 120, 170 µg/ml), polymyxin B (0.3, 0.6, 30, 60, 120, 170 µg/ml) ampicillin (0.3, 0.6, 30, 60, 120, 170 µg/ml) were utilized to carry out the present investigational work.

### Preparation of bacterial suspension and treatment

100 µl of bacterial culture was freshly inoculated in 5 ml of Mueller Hinton Broth (MHB; Hi-media) and incubated at 37 °C for 360 min, where final bacterial concentration of ~1.1 × 10^9^ colony forming units/ml (CFU/ml) was reached (mid log phase; 0.4 at OD_590_), indicating satisfactory growth of bacteria (Soon et al. 2011). The bacterial suspensions were centrifuged at 10,000 rpm, (20 min), the supernatant was discarded and the cell pellets were washed five times with 0.5 mM potassium phosphate buffer solution (pH 7.4). The bacterial cell suspension was prepared by re-suspending the cell pellet in 0.5 mM potassium phosphate buffer solution (pH 7.4). The OD_590_ of the final dispersion varied between 0.12 and 0.15 (Kłodzińska et al. 2010). The washed bacterial cell suspensions were incubated with different concentration of CTAB (0.3, 0.6, 30, 60, 120, 170 µg/ml), polymyxin B (0.3, 0.6, 30, 60, 120, 170 µg/ml) and ampicillin (0.3, 0.6, 30, 60, 120, 170 µg/ml) for different time periods (30, 60, 90 and 120 min).

### Estimation of Zeta potential

The Zeta potential depends on the composition of the cell surface as well as on the nature of the surrounding medium. Several factors such as conductivity (salt concentration) and pH of the medium govern the adsorption of ions onto bacterial cells and influence the degree of ionization of charged moieties on the cell surface (Soon et al. 2011). Keeping these in view, the Zeta potential measurements were performed with 0.5 mM potassium phosphate buffer solution (pH 7.4) to minimize any influence of pH. The Zeta potential was measured with the help of a Zetasizer Nano ZS 90 device (Malvern, UK), equipped with Helium–Neon laser (633 nm) as a source of light, with the detection at 90 degree scattering angle at room temperature (28 °C). Each of the experiments was carried out under identical experimental condition (n = 6). Zeta potential was also recorded for autoclaved (at 121 °C, at 15 psi, for 20 min) (Martinez et al. 2008) and ampicillin treated bacterial cells.

### Estimation of polydispersity index

The PDI of the samples was measured in a cuvette at 90 degree scattering angle, with the Zetasizer for both untreated and CTAB treated bacterial cells. This experiment was conducted at room temperature (28 °C).

### Determination of viability of bacterial suspension

The viability of bacteria was determined by calculating the CFU. The number of CFU was determined for the control (untreated bacterial cells) and for the bacterial cells treated with CTAB (0.3, 0.6, 30, 60, 120, 170 µg/ml), and Ampicillin (0.3, 0.6, 30, 60, 120, 170 µg/ml).

### Measurement of permeability with 1-*N*-phenylnaphtylamine (NPN)

The outer membrane permeability of *E. coli* and *S. aureus* was determined according to Helander (Helander and Mattila-Sandholm 2000). A 10 mM stock solution of NPN (in ethanol) was diluted to a concentration of 20 μM with 0.5 mM potassium phosphate buffer (pH 7.4) solution. The fluorescence of the samples was measured at an excitation and emission wavelength of 340 and 420 nm, respectively (Spectra Max M5). NPN permeability assay was also carried out for autoclaved and ampicillin treated bacterial cells.

### Measurement of permeability with crystal violet

The alteration in membrane permeability was also evaluated by crystal violet (CV) assay (Vaara and Vaara 1981; Devi et al. 2010). Bacterial cell suspensions were harvested at 4500×*g* for 5 min at 4 °C. The cells were washed twice and resuspended in 0.5 mM potassium phosphate buffer solution (pH 7.4). Washed bacterial cell suspension was incubated with CTAB and Polymyxin B at 37 °C for 30 min. Control samples were prepared similarly without treatment. The cells were resuspended in 0.5 mM potassium phosphate buffer solution (pH 7.4) containing 10 g/ml of crystal violet after harvesting them at 9300×*g* for 5 min. After incubating the suspension at 37 °C for 10 min the suspension was centrifuged at 13,400×*g* for 15 min and the OD of the supernatant was measured at a wavelength of 590 nm (SPECTRA MAX M5). OD of the supernatant of the normal untreated cell was used as blank. The OD value of crystal violet solution, was considered as 100 %. The percentage of crystal violet uptake was expressed as follows:

$$({\text{OD value of sample}}/{\text{OD value of CV solution}}) \; \times \; 100\; = \; {\text{Percentage uptake of crystal violet}}.$$

### Confocal laser scanning microscopic (CLSM) analysis

According to Stocks (2004), certain dyes can be very useful for studying the viability of cells. Substances like propidium iodide (PI) or Syto 9 are popularly used for this purpose. PI has been found to penetrate cells with damaged membranes, whereas Syto 9 is capable of penetrating the cells nonspecifically, resulting in a green fluorescence. Thus, when used simultaneously, Syto9 can replace PI and thus change the colour of the cells under fluorescence (530 nm). Two times washed bacterial cell suspension was incubated with and without CTAB 30 µg/ml. Here, the bacterial cell suspensions (both CTAB 30 µg/ml and untreated blank) were stained with 2.5 μM SYTO9 and 15 μM PI and incubated for 5 min in the dark. Cells were monitored under a confocal laser scanning microscope (CLSM; Andor spinning disk confocal microscope). The laser was used at 488 nm for excitation, and the emission was observed at 528 nm (SYTO9) and 645 nm (PI). Cells containing intact cell surface were stained fluorescent green, whereas with disrupted membranes, stained fluorescent red. The overlap of the red and green areas appears as orange (Sarkar et al. 2015). The image was analysed with ImageJ software.

### Statistical analysis

All the reported values represent the average of six independent experiments. Statistical analysis was performed with one-way analysis of variance (ANOVA) followed by post hoc Dunnett’s test. Statistical significance was defined as p < 0.05 for all tests.

## Abbreviations

CTAB: cetyl trimethyl ammonium bromide
*S. aureus*: *Staphylococcus aureus*
*E. coli*: *Escherichia coli*
MHB: Mueller–Hinton Broth
PDI: polydispersity index
CFU: colony forming unit
rpm: revolutions per minute
min: minute
OD: optical density
NPN: 1-*N*-phenylnaphtylamine
CV: crystal violet
PI: propidium iodide
CLSM: confocal laser scanning microscope
ANOVA: one-way analysis of variance
OTAP: ovotransferrin antimicrobial peptide
LPS: lipopolysaccharide
OM: outer membrane

## References

[CR1] Ahn SJ, Sivaguru M, Osawa H, Chung C, Matsumoto H (2001). Aluminum inhibits the H+-ATPase activity by permanently altering the plasma membrane surface potentials in squash roots. Plant Physiol.

[CR2] Alves CS, Melo MN, Franquelim HG, Ferre R, Planas M, Feliu L, Bardají E, Kowalczyk W, Andreu D, Santos NC, Fernandes MX, Castanho MA (2010). *Escherichia coli* cell surface perturbation and disruption induced by antimicrobial peptides BP100 and pepR. J Biol Chem.

[CR3] Andrews JM (2001). Determination of minimum inhibitory concentrations. J Antimicrob Chemoth.

[CR4] Arakha M, Saleem M, Mallick BC, Jha S (2015). The effects of interfacial potential on antimicrobial propensity of ZnO nanoparticle. Sci Rep.

[CR5] Balhara V, Schmidt R, Gorr SU, DeWolf C (2013). Membrane selectivity and biophysical studies of the antimicrobial peptide GL13K. Biochim Biophys Acta.

[CR6] Bambeke FV, Mingeot-Leclercq MP, Struelens MJ, Tulkens PM (2008). The bacterial envelope as a target for novel anti-MRSA antibiotics. Trends Pharmacol Sci.

[CR7] Berry V, Gole A, Kundu S, Murphy CJ, Saraf RF (2005). Deposition of CTAB-terminated nanorods on bacteria to form highly conducting hybrid systems. J Am Chem Soc.

[CR8] Borrok D, Fein JB, Kulpa CF (2004). Proton and Cd adsorption onto natural bacterial consortia: testing universal adsorption behavior. Geochim Cosmochim Acta.

[CR9] Chen G, Walker SL (2007). Role of solution chemistry and ion valence on the adhesion kinetics of groundwater and marine bacteria. Langmuir.

[CR10] Cieśla J, Bieganowski A, Janczarek M, Sypniewska TU (2011). Determination of the electrokinetic potential of *Rhizobium leguminosarum* bv trifolii Rt24.2 using Laser Doppler Velocimetry—a methodological study. J Microbiol Meth.

[CR11] Clark RH, Campbell AA, Klumb LA, Long CJ, Stayton PS (1999). Protein electrostatic surface distribution can determine whether calcium oxalate crystal growth is promoted or inhibited. Calcif Tissue Int.

[CR12] Devi KP, Nisha SA, Sakthivel R, Pandian SK (2010). Eugenol (an essential oil of clove) acts as an antibacterial agent against *Salmonella typhi* by disrupting the cellular membrane. J Ethnopharmacol.

[CR13] Domingues MM, Inácio RG, Raimundo JM, Martins M, Castanho MARB, Santos NC (2012). Biophysical characterization of polymyxin b interaction with LPS aggregates and membrane model systems. Pept Sci.

[CR14] Domingues MM, Silva PM, Franquelim HG, Carvalho FA, Castanho MA, Santos NC (2014). Antimicrobial protein rBPI21-induced surface changes on Gram-negative and Gram-positive bacteria. Nanomedicine.

[CR15] Eboigbodin KE, Newton JR, Routh AF, Biggs CA (2006). Bacterial quorum sensing and cell surface electrokinetic properties. Appl Microbiol Biotechnol.

[CR16] Falagas ME, Kasiakou SK (2005). Colistin: the revival of polymyxins for the management of multidrug-resistant Gram-negative bacterial infections. Clin Infect Dis.

[CR17] Fernandes HP, Cesar CL, Barjas-Castro ML (2011). Electrical properties of the red blood cell membrane and immunohematological investigation. Rev Bras Hematol Hemoter.

[CR18] Giuliani A, Pirri G, Bozzi A, Giulio A, Aschi M, Rinaldi AC (2008). Antimicrobial peptides: natural templates for synthetic membrane-active compounds. Cell Mol Life Sci.

[CR19] Helander IM, Mattila-Sandholm T (2000). Fluor metric assessment of Gram-negative bacterial permeabilization. J Appl Microbiol.

[CR20] Herben PFG, Mozes N, Rouxhet PG (1990). Variation of the surface properties of *Bacillus licheniformis* according to age, temperature and aeration. Biochim Biophys Acta.

[CR21] Hurdle JG, O’Neill AJ, Chopra I, Lee RE (2011). Targeting bacterial membrane function: an under exploited mechanism for treating persistent infections. Nat Rev Microbiol.

[CR22] Ibrahim HR, Sugimoto Y, Aoki T (2000). Ovotransferrin antimicrobial peptide (OTAP-92) kills bacteria through a membrane damage mechanism. Biochim Biophys Acta.

[CR23] Jucker BA, Harms H, Hug SJ, Zehnder AJB (1997). Adsorption of bacterial surface polysaccharides on mineral oxides is mediated by hydrogen bonds. Coll Surf B Biointerfaces..

[CR24] Katz M, Tsubery H, Kolusheva S, Shames A, Fridkin M, Jelinek R (2003). Lipid binding and membrane penetration of polymyxin B derivatives studied in a biomimetic vesicle system. Biochem J.

[CR25] Kennedy D, Cronin UP, Wilkinson MG (2011). Responses of *Escherichia coli*, *Listeria monocytogenes*, and *Staphylococcus aureus* to simulated food processing treatments, determined using fluorescence-activated cell sorting and plate counting. Appl Environ Microbiol.

[CR26] Kłodzińska E, Szumski M, Dziubakiewicz E, Hrynkiewicz K, Skwarek E, Janusz W, Buszewski B (2010). Effect of zeta potential value on bacterial behavior during electrophoretic separation. Electrophoresis.

[CR27] Li Y, Xiang Q, Zhang Q, Huang Y, Su Z (2012). Overview on the recent study of antimicrobial peptides: origins, functions, relative mechanisms and application. Peptides.

[CR28] Loh B, Grant C, Hancock RE (1984). Use of the fluorescent probe 1-*N* phenylnaphthylamine to study the interactions of aminoglycoside antibiotics with the outer membrane of *Pseudomonas aeruginosa*. Antimicrob Agents Chemother.

[CR29] Loosdrecht MCV, Lyklema J, Norde W, Schraa G, Zehnder AJ (1987). Electrophoretic mobility and hydrophobicity as a measured to predict the initial steps of bacterial adhesion. Appl Environ Microbiol.

[CR30] Maillard JY (2002). Bacterial target sites for biocide action. J Appl Microbiol Symp Suppl.

[CR31] Martinez RE, Pokrovsky OS, Schott J, Oelkers EH (2008). Surface charge and zeta potential of metabolically active and dead cyanobacteria. J Colloid Interf Sci.

[CR32] Mendes CAC, Burdmann EA (2010). Polymyxin – a review focusing on their nephrotoxicity. Rev Assoc Med Bras.

[CR33] Mills AL, Herman JS, Hornberger GM, DeJesus TH (1994). Effect of ionic strength and iron coatings on mineral grains on sorption of bacterial cells to quartz sand. Appl Environ Microbiol.

[CR34] Nguyen LT, Haney EF, Vogel HJ (2011). The expanding scope of antimicrobial peptide structures and their modes of action. Trends Biotechnol.

[CR35] Nikaido H, Neidhardt FC, Curtiss IIIR, Ingraham JL, Lin ECC, Low KB, Magasanik B, Reznikoff WS, Riley M, Schaechter M, Umbarger HE (1996). Outer membrane. *Escherichia coli* and *Salmonella*: cellular and molecular biology.

[CR36] Noller HF, Cate J, Dallas A, Culver G, Earnest TN, Green R, Holmberg L, Joseph S, Lancaster L, Lieberman K, Merryman C, Newcomb L, Samaha R, Ahsen VU, Yusupov M, Yusupova G, Wilson K, Garrett RA, Douthwaite SR, Liljas A, Matheson AT, Moore PB, Noller HF (2000). Studies on the structure and function of ribosomes by combined use of chemical probing and X-ray crystallography. The ribosome: structure, function, antibiotics, and cellular interactions.

[CR37] Payne DJ, Gwynn MN, Holmes DJ, Pompliano DL (2007). Drugs for bad bugs: confronting the challenges of antibacterial discovery. Nat Rev Drug Discov.

[CR38] Powers JP, Hancock RE (2003). The relationship between peptide structure and antibacterial activity. Peptides.

[CR39] Saito T, Takatsuka T, Kato T, Ishihara K, Okuda K (2001). Adherence of oral streptococci to an immobilized antimicrobial agent. Arch Oral Biol.

[CR40] Sarkar R, Mondal C, Bera R, Chakraborty S, Barik R, Roy P, Kumar A, Yadav KK, Choudhury J, Chaudhary SK, Samanta SK, Karmakar S, Das S, Mukherjee PK, Mukherjee J, Sen T (2015). Antimicrobial properties of *Kalanchoe blossfeldiana*: a focus on drug resistance with particular reference to quorum sensing-mediated bacterial biofilm formation. J Pharm Pharmacol.

[CR41] Sato H, Feix JB (2006). Peptide-membrane interactions and mechanisms of membrane destruction by amphipathic alpha-helical antimicrobial peptides. Biochim Biophys Acta.

[CR42] Scholl MA, Harvey RW (1992). Laboratory investigations on the role of sediment surfaces and ground water chemistry in transport of bacteria through a contaminated sandy aquifer. Environ Sci Technol.

[CR44] Sharma PK, Rao KH (2003). Adhesion of *Paenibacillus polymyxa* on chal copyrite and pyrite: surface thermodynamic and extended DLVO approaches. Colloids Surf B Biointerfaces.

[CR45] Simões M, Pereira MO, Vieira MJ (2005). Action of a cationic surfactant on the activity and removal of bacterial biofilms formed under different flow regimes. Water Res.

[CR46] Sondi I, Salopek-Sondi B (2005). The influence of primary structure of enzymes on the formation of CaCO_3_ polymorphs: a comparison of plant (*Canavalia ensiformis*) and bacterial (*Bacillus pasteurii*) ureases. Langmuir.

[CR47] Soon RL, Nation RL, Cockram S, Moffatt JH, Harper M, Adler B, Boyce JD, Larson I, Li J (2011). Different surface charge of colistin-susceptible and -resistant *Acinetobacter baumannii* cells measured with zeta potential as a function of growth phase and colistin treatment. J Antimicrob Chemother.

[CR48] Stocks SM (2004). Mechanism and use of the commercially available viability stain, BacLight. Cytometry.

[CR49] Strahl H, Hamoen LW (2010). Membrane potential is important for bacterial cell division. PNAS.

[CR50] Takashima S, Morisaki H (1997). Surface characteristics of the microbial cell of *Pseudomonas syringae* and its relevance to cell attachment. Colloids Surf B Biointerfaces.

[CR51] Tokumasu F, Ostera GR, Amaratunga C, Fairhurst RM (2012). Modifications in erythrocyte membrane zeta potential by *Plasmodium falciparum* infection. Exp Parasitol.

[CR52] Torcato M, Castanho MARB, Henriques ST (2012). The application of biophysical techniques to study antimicrobial peptides. Spectrosc Int J.

[CR53] Torcato IM, Huang YH, Franquelim HG, Gaspar DD, Craik DJ, Castanho MA, Henriques ST (2013). The antimicrobial activity of Sub3 is dependent on membrane binding and cell-penetrating ability. Chembiochem.

[CR54] Tsuchido T, Katsui N, Takeuchi A, Takano M, Shibasaki I (1985). Destruction of the outer membrane permeability barrier of *Escherichia coli* by heat treatment. Appl Environ Microbiol.

[CR55] Urrutia-Mera M, Kemper M, Doyle R, Beveridge TJ (1992). The membrane-induced proton motive force influences the metal binding ability of *Bacillus subtilis* cell walls. Appl Environ Microbiol.

[CR56] Vaara M, Vaara T (1981). Outer membrane permeability barrier disruption by Polymyxin in Polymyxin-susceptible and -resistant *Salmonella typhimurium*. Antimicrob Agents Chemother.

[CR57] Walker SL, Hill JE, Redman JA, Elimelech M (2005). Influence of growth phase on adhesion kinetics of *Escherichia coli* D21g. Appl Environ Microbiol.

[CR58] Wiese A, Munstermann M, Gutsmann T, Lindner B, Kawahara K, Zahringer U, Seydel U (1998). Molecular mechanisms of Polymyxin B-membrane interactions: direct correlation between surface charge density and self-promoted transport. J Mem Biol.

[CR59] Wilson WW, Wade MM, Holman SC, Champlin FR (2001). Status of methods for assessing bacterial cell surface charge properties based on zeta potential measurements. J Microbiol Methods.

[CR60] Yee N, Fein JB, Daughney CJ (2000). Experimental study of the pH, ionic strength and reversibility behavior of bacteria-mineral adsorption. Geochim Cosmochim Acta.

[CR61] Yongsuk H, Brown DG (2006). Cell surface acid–base properties of *Escherichia coli* and *Bacillus brevis* and variation as a function of growth phase, nitrogen source and C:N ratio. Colloids Surf B Biointerfaces.

[CR62] Yongsuk H, Brown DG (2008). Electrostatic behavior of the charge-regulated bacterial cell surface. Langmuir.

[CR63] Yoshinari M, Oda Y, Kato T, Oukda K, Hirayama A (2000). Influence of surface modifications to titanium on oral bacterial adhesion in vitro. J Biomed Mater Res.

